# The use of innovative approaches to strengthen health system resilience during the COVID-19 pandemic: case studies from selected Commonwealth countries

**DOI:** 10.3389/fpubh.2023.1115415

**Published:** 2023-04-27

**Authors:** Janneth Mghamba, Emily Gilmour, Layne Robinson, Azma Simba, Albert Tuyishime, Anand Persaud, Charles Mwansambo, Lakshmi Somatunga, Solomon Werema, Witness Mchwampaka, Vida Makundi, Kakulu Remedius, Fidelis Ronjiono, Beatrice Mutayoba, Theophile Dushime, Edison Rwagasore, Baptiste Byiringiro, Sylvere Mugumya, Claude Muvunyi, Frank Anthony, Narine Singh, Joseph Tsung-Shu Wu, Simeon Yosefe, Queen Dube, Nimdinu Mayakaduwa, Rangana Wadugedara

**Affiliations:** ^1^Commonwealth Secretariat, London, United Kingdom; ^2^Ministry of Health, Community Development, Gender, Elderly and Children (Tanzania), Dar es Salaam, Tanzania; ^3^Ministry of Health (Rwanda), Kigali, Rwanda; ^4^Ministry of Health Guyana, Georgetown, Guyana; ^5^Ministry of Health (Malawi), Lilongwe, Malawi; ^6^Ministry of Health Sri Lanka, Colombo, Sri Lanka; ^7^Rwanda Biomedical Center, Kigali, Rwanda

**Keywords:** best practices, COVID-19 response, Commonwealth, digital innovations, multisectoral engagement, health system resilience

## Abstract

This article is part of the Research Topic ‘Health Systems Recovery in the Context of COVID-19 and Protracted Conflict’.

The COVID-19 pandemic has exposed the vulnerabilities and limitations of many health systems and underscored the need for strengthening health system resilience to make and sustain progress toward Universal Health Coverage (UHC), global health security and healthier populations in tandem. In response to the COVID-19 pandemic, Commonwealth countries have been practicing a combination of innovative integrated approaches and actions to build health systems resilience. This includes utilizing digital tools, improvements in all-hazard emergency risk management, developing multisectoral partnerships, strengthening surveillance and community engagement. These interventions have been instrumental in strengthening national COVID-19 responses and can contribute to the evidence-base for increasing country investment into health systems resilience, particularly as we look toward COVID-19 recovery. This paper gives perspectives of five Commonwealth countries and their overall responses to the pandemic, highlighting practical firsthand experiences in the field. The countries included in this paper are Guyana, Malawi, Rwanda, Sri Lanka, and Tanzania. Given the diversity within the Commonwealth both in terms of geographical location and state of development, this publication can serve as a useful reference for countries as they prepare their health systems to better absorb the shocks that may emerge in future emergencies.

## Introduction

The impact of the COVID-19 pandemic has been widely felt around the world – and Commonwealth countries have not been spared (1, 2). The Commonwealth, which is a voluntary association of 56 independent countries spread across Africa, Asia, the Americas, Europe and the Pacific, has experienced major economic and health crises as a result of the COVID-19 pandemic, with over 107 M cases out of a total of 760 M globally (3).

From an economic standpoint, Commonwealth countries collectively lost as much as US$475 billion worth of exports in 2020, including $100 billion in intra-Commonwealth trade. Intra-Commonwealth exports rebounded in 2021 and are estimated to have reached $768 billion, the highest recorded in value terms (4). Likewise, the pandemic has left even the most well-equipped health systems in the Commonwealth vulnerable and has stalled progress toward wider global and national health priorities, including Universal Health Coverage and the Sustainable Development Goals (SDGs). In 2023, the Commonwealth remains far short of reaching the SDG target of 3.8 to achieve UHC (5). Commonwealth leaders sought to address this at the last Commonwealth Heads of Government Meeting in 2022, and “recognized the importance of strong, resilient, and inclusive health systems … for countries to better prepare, prevent, detect, respond and recover from health emergencies” (6).

The COVID-19 pandemic has demonstrated that health system resilience – defined as the health system’s ability to prepare for, resist, manage, adapt, recover, and learn from a hazard and its effects – is key in maintaining and strengthening health system functionality (7). Countries around the globe are confronting the challenge of how to recover from the legacies of the pandemic, which provides an opportunity to learn from countries grappling with common challenges and asking similar questions about what a resilient health system should look like. Some of these examples include leveraging health information for immediate and robust actions, whole-of-society approaches and a recognition of the interconnectedness of health systems and public health emergency management.

We present here perspectives from five Commonwealth countries: Guyana, Malawi, Rwanda, Sri Lanka and Tanzania. While there is no one size fits all approach, the lessons derived in this publication can aid in understanding how resilient health systems can be developed and operationalized in different contexts.

### Method

In recognition of the need to understand what worked and what did not work during the COVID-19 pandemic, the Health Section in the Commonwealth Secretariat contacted officials within Commonwealth countries to propose a compendium of case studies on lessons learnt from the pandemic. Representatives from the five Ministries of Health participating in this publication include Ministers of Health, Permanent Secretaries, Chief Medical Officers, National Focal Points for COVID-19, Epidemiologists, and other key decision makers in national COVID-19 response plans. The participation of senior Ministry of Health representatives was key given their unique position to comment on the development, implementation and evaluation of national COVID-19 responses. Country officials were requested to submit relevant examples of lessons learnt from the COVID-19 pandemic, under three broad themes: digital health, pandemic management and maintaining essential health services. These case studies were then developed and integrated by the Commonwealth Secretariat in collaboration with Ministries of Health.

Supplementary information was sourced through national documents and reports, literature reviews of peer reviewed publications, data sourced from World Health Organization and other international health agencies, and government webpages. For each country, data from March 2020 to September 2022 were collected.

## Country responses for effective COVID-19 pandemic management

### Guyana case study: Strengthening COVID-19 governance through establishing a national COVID-19 taskforce

Guyana’s National COVID-19 Taskforce, which is the focus of this case study, has been instrumental in mounting an integrated response strategy since the first case of COVID-19 was identified on 11 March 2020. Between March 11, 2020, and September 1, 2022, Guyana had 87,835.57 of cumulative confirmed COVID-19 cases per million people and 1,570.26 confirmed deaths per million people (3). In the early stages of the COVID-19 pandemic, the coping capacity of Guyana’s healthcare system was limited in responding to health emergencies. While in the history of Guyana, no such epidemic or health event of this magnitude has occurred, there have been smaller outbreaks or suspected outbreaks in different locations of the country, which usually garnered a response mainly from the Ministry of Health. As a single entity, the Ministry of Health’s capacity for responding to the COVID-19 outbreak was severely limited and therefore identifying effective approaches to coordinate the mitigation of cases while retaining public confidence was key.

The country established the National COVID-19 Task Force and 10 subordinate committees for each of the administrative regions of Guyana, to oversee the coordination and implementation of the COVID-19 pandemic response strategy (8). The Taskforce used an integrated approach focused on strong leadership and state and non-state partnership which corresponds with successful interventions outlined in existing literature. The National COVID-19 Task Force was led by the Prime Minister and had the participation of ministers, directors and leaders representing health, disciplined services, civil defense, tourism, finance, trade, commerce, points of entry, immigration, law enforcement, private sector commissions, religious leaders, indigenous leaders, non-governmental agencies, etc. As a result, the Taskforce became the largest assembly of state and non-state representatives working together and sharing resources to achieve a shared goal. Ten Regional COVID-19 Task Force committees had a similar composition and conducted activities at the level of their respective administrative regions. This multisectoral approach ensured that all support, in every aspect, was directed to COVID-19 response.

The accumulated resources dispensed by the state and non-state partnership were utilized to address many issues, including to procure and implement a free COVID-19 vaccination program for all persons within Guyana, implement subsidies on food items and basic hygiene products and provide free primary, secondary and tertiary healthcare services to its population.

One of the most logistically challenging measures was the implementation of the National COVID-19 Vaccination Program, which included transporting and storing vaccines in line with cold-chain requirements to distant and peripheral regions of Guyana and sensitizing the public and responding to vaccine hesitancy. However, due to the support and use of resources from state agencies, the private sector and non-state leaders through the Taskforce, Guyana was able to operate almost 150 fixed and mobile vaccination sites daily in all 10 administrative regions, with vaccination teams working on weekends and holidays and visiting hard-to reach communities and ensuring service provision to all eligible persons free of cost regardless of citizenship.

The success of this program is reflected in Guyana’s COVID-19 vaccination coverage, with the first national dose coverage in the adult population reaching 87.6 percent and the second dose covering 68.2 percent (3). The Government also took measures to ensure that the rights and privileges of both citizens and migrants were not infringed upon. Migrants originating from neighboring Venezuela were afforded special considerations to safeguard their health and safety, as their circumstances rendered them among the most vulnerable. This was considered an important part of the mitigation strategy as there were approximately 33,000 Venezuelan migrants in Guyana.

Another key priority for the National COVID-19 Taskforce was to ensure that essential health services at the primary, secondary and tertiary level were maintained. Efforts included the Ministry of Health’s creation of a Package of Essential Health Care Services for Primary Health Care in March 2022, with 215 different health interventions to be offered at every health institution in the country (9). The Task Force made use of support from other state agencies and healthcare professionals to provide primary healthcare services to all citizens within their catchment area including specialized clinics for maternal and child health, pediatric clinics, and NCD clinics. Emergency and surgical interventions continued based on the level of urgency while non-essential services were either postponed or conducted through home visits.

Through the implementation of the National COVID-19 Taskforce, which remained operational until the end of 2022, Guyana was able to pool together sufficient human, material, financial, logistical, and other resources to mitigate the impact of the pandemic. Beyond COVID-19, this institutional mechanism will be key to address future preparedness and response in health shocks and emergencies.

### Malawi case study: Adopting resiliency approaches for national preparedness and response

The COVID-19 pandemic response in Malawi was aided by its Ministry of Health’s existing frameworks for health emergency preparedness and response, which will be the focus of this case study. By the time the first three cases were detected on April 2, 2022, and through the COVID-19 peaks, with a cumulative number of recorded cases and deaths per million at 4,306.57 and 139.09, respectively, between then and September 1, 2022, Malawi was better positioned to mitigate the spread and impact of the pandemic (3).

In 2019, Malawi conducted the first Joint External Evaluation (JEE) of International Health Regulation (IHR) core capacities (10). During this evaluation, the country’s capacity for public health emergency preparedness and response was highlighted for improvement. Based on these findings, the major challenges affecting pandemic management in Malawi were: (1) inadequate Incident Management System (IMS) capacity, including human and infrastructure challenges at national and district levels for planning, emergency detection, coordination and responses; (2) lack of fully functional emergency operations centers (EOCs) and an operational hotline for handling a disease of unknown origin; and (3) a national multi-hazard contingency plan which does not address emergency preparedness for IHR-relevant hazards, including those that have the potential to cause Public Health Emergencies of International Concern (PHEIC). Before the pandemic, Malawi adopted a One Health approach for the country’s epidemic preparedness and response, listed in the Health Sector Strategic Plan II 2017–2022 (HSSP II) as one mechanism to address the gaps identified by JEE. An online instant message (IM) forum was created to gather all one health related stakeholders together, and a “One Health Surveillance Platform (OHSP)” was established in 2019. The OHSP was developed using the open-source district health information system 2 (DHIS2) technology and aligned with the open health information exchange (OpenHIE) framework to accommodate country “One Health” surveillance needs from human, animal and environmental domains. The IM and OHSP platforms were applied to enhance outbreak and emergency detection, and coordination for preparedness. All interventions were established before the COVID-19 pandemic to better prepare for potential future health shocks and emergencies. To support these interventions, the Ministry of Health initiated the development of a National Action Plan for Health Security (NAPHS) in collaboration with the Department of Disaster Management Affairs. The priority focus of these interventions was to increase Malawi’s health system resiliency when confronted by a potential epidemic or pandemic in the future, which provided useful when the IM/OHSP picked up the alert for the start of the COVID-19 outbreak. Following the formal declaration of a PHEIC by the World Health Organization in March 2020, the government put in place a state of disaster in the country and installed several preventive measures to mitigate its severity.

After Malawi registered its first cases, the Ministry of Health activated its national-level COVID-19 Emergency Operations Centre (EOC) the following day to ensure UHC efforts were not disrupted by COVID-19 and to coordinate and execute all COVID-19 response activities, including but not limited to surveillance, contact tracing, border health, clinical care and treatment, risk communication and community engagement. The EOC set up several initiatives, including launching a dedicated 24/7 hotline to receive public incidents, as well as various digital tools such as RapidPro, a community toolkit for health education and public communication, and EOC internal dashboards to manage the pandemic effectively (11). Regarding the EOC hotline and call center operations, the Ministry of Health harmonized the health-related hotlines by combining the Chipatala cha pa Foni (CCPF), a ministry-owned telehealth service, and the rapidly established COVID-19 EOC hotline into one call number (929). The calls were centrally monitored and responded by the EOC call center operators (12, 13). Between the establishment of the center in early 2020 to its closure in July 2022, the call center received 2,929,984 calls, including self-suspect reporting, COVID-19-related information checking, vaccination and digital certificate inquiries, and adverse event after immunization reporting.

The response strategies exemplified in this case study demonstrate some key indicators for successful pandemic response, including steering the response through effective, timely and comprehensive systems, and utilizing a range of channels to engage and include the country population in mitigating the spread of COVID-19. Of particular focus is Malawi’s Emergency Operations Center (EOC), which served as the data-driven core of the government’s pandemic response to coordinate pandemic responses across the country, and which leveraged routine and novel data sources to address the rapidly evolving pandemic. Malawi’s efforts to adequately prepare for health emergencies can provide insights into the linkages between pandemic preparedness and response.

### Rwanda case study: Leveraging existing digital health technologies to strengthen national COVID-19 testing and vaccinations

Below we highlight the interventions made by Rwanda to utilize digital solutions to mitigate the spread of COVID-19. Rwanda has made significant progress in recent years toward its goal of becoming a middle-income country by 2035 and a high-income country by 2050. Rwanda’s development is supported by strong government investment in the country’s digital transformation, digital government systems and digital connectivity to increase affordability and access. The Rwanda Health Management Information System (HMIS) was established in 1998 with the goal to improve the quality of routinely collected health data from community health workers and the system has been upgraded to a web-based system known as the District Health Information System Version 2 (DHIS2) (14).

While Rwanda had taken steps to be better prepared for health emergencies, like many countries, it faced challenges in responding to the COVID-19 pandemic, including limited capability to receive national COVID-19 statistics data, delays in laboratory results and lack of digital solutions to facilitate cross border travelers. When the first case was detected on March 14, 2020, the operational response to the COVID-19 pandemic required the rapid adaptation and leveraging of the existing HMIS to collect, transmit and analyze key health data in real-time to increase understanding of the epidemiological situation and support in designing appropriate control measures (3). Rwanda, as one of the more advanced countries in promoting information technology in the region, maintained focus on applying technologies for the surveillance and control of the COVID-19 pandemic (15). The establishment of the national command post also played a key role in coordinating COVID-19 surveillance and the digital solutions (16). The command post facilitated rapid deployment of digital solutions utilizing the existence of the national strategy and pre-existing infrastructure.

The digital solutions developed by Rwanda during the COVID-19 pandemic emphasize patient access, enabling individuals to directly receive or track their own test results. It also minimizes the strain on the health sector to communicate results and to issue COVID-19 test result certificates where needed through the integration of laboratory and health management information systems across the cascade of COVID-19 diagnosis. The use of mobile data collection tools for community-based surveillance generated valuable insights to inform timely responses to outbreaks. Tracing and monitoring of cases and contacts using digital tools reduced the burden on the health system and allowed the country to focus its limited capacity on delivering services to the most at-risk individuals.

The system reports real-time data. For instance, the system has been able to report COVID-19 cases since the first case was detected. Between March 14, 2020, and September 1, 2022, the cumulative number of confirmed COVID-19 cases and deaths per million people is reported as 9,612.02 and 106.41, respectively, (3). The system to monitor COVID-19 was able to handle multiple concurrent users up to 9,000 in vaccination and more than 3,200 in a Covid-19 testing environment. With the existing digital tools, COVID-19 cases in the community have been monitored and provided with communication to report on their status.

Through leveraging existing HMIS technology, Rwanda gained increased capability to provide the required rapid response to the pandemic in areas of surveillance and contact tracing, case management, and in maintaining access to high-quality essential services. The collaboration between multiple arms of government and the private sector facilitated the deployment of these digital solutions through enabling the health sector to leverage existing data systems. These digital solutions led to a greater degree of health system resiliency, particularly through increased testing capacity and clinical management.

### Sri Lanka case study: Interventions to maintain essential health services during the COVID-19 pandemic

Given Sri Lanka’s position as one of the first countries globally to commit to working toward Universal Health Coverage, it is helpful to understand how it modified healthcare delivery to ensure pre-existing health services were adequately maintained (5). The first case of the virus in Sri Lanka was confirmed on 27 January 2020. Sri Lanka opted for a containment strategy like that in Singapore (17). In view of global disease trends and patterns, the health authorities focused on strengthening the hospital emergency preparedness and response plans of all health-care institutions. The emerging needs of these institutions were addressed by the government using a three-tier approach: (1) declaration of designated COVID-19 treatment facilities, (2) declaration of isolation hospitals and (3) identification of centers with ICU/HDU facilities in the country (18). While many countries had challenges on planning for essential health services, Sri Lanka ensured that special measures were in place for continued services for routine care while managing COVID-19. National guidelines were developed for the management of noncommunicable diseases (NCDs) and other routine clinics at hospitals and care arrangements for vulnerable groups. The government also worked under a whole of society approach through which the non-health sectors cooperated and were involved in supporting infrastructure facilities, mobility and providing their vehicles and equipment for the distribution of essentials and medicines (19). The state military and police extended support in contact tracing, quarantine measures and vaccine drives, reflecting the commitment to a Whole of Government and Whole of Society approach to COVID-19 in Sri Lanka (20).

In response to the COVID-19 lockdown measures, the country instituted modified means of healthcare delivery to ensure continuity of health services. During the planning phase, the Ministry of Health developed and disseminated guidelines using electronic media, for the smooth continuation of essential services related to maternal and child health (MCH) services in both the curative and preventive sector (21). Small scale alternative clinics targeting several clusters were established so that both parents and children could walk to nearby outreach center for vaccination. The Family Health Bureau, which operates under the Ministry of Health, released specific guidelines to ensure uninterrupted field maternal and child health-care services for lockdown areas and quarantined families (18).

Throughout the pandemic, the epidemiological information was shared continuously, and the weekly epidemiological situation by WHO was of immense importance and thereby evidence-based policy decisions were possible to be made. For instance, between March 2020 and September 1, 2022, the cumulative number of confirmed COVID-19 cases per million people was recorded as 30,692.21 and confirmed cumulative deaths per million people was 764.88 (3). This surveillance information has been used to devise surveillance strategies with the rapid spread of infection seen during the third wave and the availability of the rapid antigen tests and for updating the testing strategy for workplaces in May 2021 (22).

The three-tiered approach used by Sri Lanka to strengthen the hospital emergency preparedness and response plans health-care institutions generated fruitful results. As of 2022, the mortality rate for COVID-19 in Sri Lanka is at 0.48 percent which is considerably lower than the global rate of 2.14 percent (23). The adaption of service delivery and the provision of alternative patient care pathways and interventions was a means of managing the treatment of COVID patients and maintaining essential non-COVID care. These effective approaches are recommended for smooth continuation of healthcare services and can inform health systems looking to build greater resilience in post-COVID recovery.

### Tanzania case study: The use of digital tools in enhancing disease surveillance measures

Tanzania’s national efforts to integrate digital products into its COVID-19 response is the focus of this case study, through its application in COVID-19 surveillance. The first COVID-19 case in Tanzania was reported on March 16, 2020. By September 1, 2022, the cumulative number of confirmed COVID-19 cases and deaths per million people was 594.69 and 12.90, respectively, (3). The COVID-19 pandemic prompted an unprecedented response from all levels of government in the country, which subsequently led to the country opting to use a mitigation strategy which focused on reducing transmission rates. This type of control strategy has also been used by other countries like the United States (24) and Italy (25). In the early stage of the pandemic, Tanzania used its existing electronic integrated disease surveillance and response system (eIDSR) to enact this strategy, which enabled initial cases that were presented at health facilities to be easily captured (26). As cases increased, the IDSR system needed to adapt to the fast-changing crisis to effectively capture cases in the community, as well as in health facilities. For those entering Tanzania at a formal point of entry, a web-based application known as Pima – meaning “measures” – was developed to enable reporting and screening. To facilitate health declarations, bookings, and rapid antigen test payments for travelers upon arrival, the government developed a travelers digital surveillance system known as Afyamsafiri meaning “Health Traveller.” These systems were both linked to the eIDSR system. In addition, linkages between point of entry screening and health service delivery systems were enhanced using a standard operating procedure which was developed to facilitate referral system. At health facilities, digital applications for COVID-19 were developed in partnership with University of Dar es Salaam to improve case base reporting at health facilities and facilitate contact tracing (27).

These digital health tools have been anchored within the District Health Management System 2 software (DHSI2) with several new indicators added to facilitate planning for surge capacity (28). These indicators included the number of individuals vaccinated against COVID-19, the number of ICU beds occupied, number of oxygen equipment and the number of health care workers infected. The platform has also been incorporated within HIV/AIDS clinics and care treatment systems to facilitate the monitoring of COVID-19 vaccinations.

This COVID-19 digital ecosystem which was integrated into the existing national surveillance has become an essential element of building resilience as it has facilitated better data-driven planning and decision-making. An interactive dashboard within the application has generated case-list reports and has enabled the country in planning for case management, contact tracing, coordination and operations, diagnostic tools, event-based surveillance, health facility and provider administration, laboratory systems, points of entry, risk communication and community engagement, routine surveillance, supply chain (29). The use of innovative digital technology in strengthening monitoring, surveillance and early warning systems can therefore be identified as a key consideration in pandemic recovery plans.

## Summary of country interventions

This publication highlights five country experiences and identifies interventions that have proved critical in responding to the COVID-19 pandemic and increasing national health system resilience. The following section attempts to synthesize some of the key learnings and interventions from these case studies.

### Governance and multisectoral approach

In the context of Guyana, Malawi, and Rwanda, the early centralized governance structure and coordination mechanisms stood out as key strategic interventions during the early COVID-19 response. Although the organization of such mechanisms varied from country to country, case studies demonstrated the need for actors from across government and in multiple sectors to be focused on one unified response plan. Indeed, the COVID-19 pandemic has offered decision-makers an opportunity to work collectively in crisis for effective planning and coordination. There are many testimonies documenting that to have an effective response, concerted multisectoral efforts involving public, private, and civil society actors within and beyond the health sector is required (30). For coordinated action to be sustainable, there is a need to have supporting structures like formalized institutional arrangements and policies which stipulate clear processes for working together. Other countries beyond the scope of this paper have demonstrated how this can be done (31). Given the interconnected nature of societal health, this level of engagement, if sustained, will be crucial to address other global health crises including the climate crisis.

### Health information system: Linking data sources and systems to identify unmet needs for essential health care

Health Management Information System (HMIS) is considered as one of the main building blocks of health systems by the World Health Organization (WHO). Health systems strengthening and efforts toward health security need to be integrated to promote sustainability, efficiency, and effectiveness at both national and subnational level. Strong HIS allow for a coordinated response in times of public health crisis and thus implicitly bear a large potential for overall economic and social benefits (32). WHO recommends having “expanded (dual) dashboard of service coverage and delivery indicators and the use of key tracer indicators on utilization patterns and mortality on both COVID-19 and non-COVID-19 conditions to manage a dual-track health system” (33). Tanzania and Rwanda case studies have shown that investment in HMIS assisted in ensuring health system resiliency. Their experiences have left a key message that integrated data reporting systems if well-built can support fine-tuning of containment measures during a pandemic as well as in recovery phases.

### Digital health: A tool for ensuring continuity of essential health services

Digital technologies have been instrumental in improving county responses to infectious-disease threats as well as in strengthening primary healthcare. All five countries embraced digital health tools to tackle a range of issues, include border surveillance, contact tracing, laboratory results and the provision of virtual patient care. In this publication, Malawi’s One Health approach to its digital tools aided the country to have a resilient information system during its COVID-19 response. Tanzania strengthened its digital health system by leveraging existing platforms and integrating COVID-19 into routine HIV/AIDS Care and Treatment Clinics. Similar examples were evident in Sri Lanka’s case study. While it is widely recognized that technologies like the Internet of Things (IoT), big data, artificial intelligence, block chain will have an impact on public health strategies, scaling up digital health will require significant institutional support to build country capabilities (34).

### Maintaining essential health services during the COVID-19 pandemic

The impact of the COVID-19 pandemic on essential health services has been demonstrated widely (35). All five countries adopted strategies to ensure essential health services were maintained and any previous progress on both communicable and noncommunicable diseases was not lost. This included the adoption of special measures for the continuation of routine care in Sri Lanka, leveraging existing digital technology to provide rapid and later incorporated vaccination in their National Response Plans in Rwanda and Tanzania, and creating a multi-sectoral response to the COVID-19 pandemic in Guyana and Malawi, bringing together actors including the private sector to maintain essential health services. Similar examples have been documented in cross-country comparisons on planning services, managing cases, and maintaining essential health services (36, 37).

## Study limitations

This paper could be strengthened through a more comprehensive review of country interventions across the Commonwealth before and during the COVID-19 pandemic. While several thematic similarities emerged across the five participating countries, the inclusion of more countries in this review would increase its rigor and understanding of the Commonwealth’s broader response to the COVID-19 pandemic. It would also be helpful to understand how these responses compare to interventions made during previous health shocks or emergencies, to provide a form of comparison. A stronger quantitative approach could also strengthen the discussions in the paper, to assess the outcome of the documented interventions more effectively.

## Conclusion

The paper has offered perspectives on country experiences in responding effectively to the COVID-19 pandemic and includes interventions that aimed to maintain essential health services, build health system resilience, and strengthen country preparedness. As countries continue to recover from the impact of the COVID-19 pandemic, these case studies present us with an opportunity to gain experience on what has worked, and what has not. The experiences of country representatives from Guyana, Malawi, Rwanda, Sri Lanka, and Tanzania, who have served as co-authors for this paper, have provided a unique observation on the impact of the discussed interventions in responding to the pandemic and in increasing health system resilience within the country.

It is hoped that these case studies, while limited in scope and size, can contribute to the broader literature to understand what is needed to strengthen health system resilience to future shocks in the spirit of building back better. The case studies call for strong leadership and governance to prioritize and invest in well-resourced health systems, including through strengthening surveillance systems, facilitating multisectoral approaches to health, implementing innovative tools such as digital technologies and incorporating strong primary health care.

The COVID-19 pandemic has made a clear case for greater investment into health and looking forward, policymakers should explore how interventions such as those discussed in this paper can support in the building of strong and resilient health systems for recovery from the pandemic and to face future health threats.

## Data availability statement

The raw data supporting the conclusions of this article will be made available by the authors, without undue reservation.

## Author contributions

JM, EG, and LR were responsible for the concept, structure and finalization of this publication. All other authors ultimately contributed sufficiently and meaningfully to the drafting and editing of the final version for submission.

## Conflict of interest

The authors declare that the research was conducted in the absence of any commercial or financial relationships that could be construed as a potential conflict of interest.

## Publisher’s note

All claims expressed in this article are solely those of the authors and do not necessarily represent those of their affiliated organizations, or those of the publisher, the editors and the reviewers. Any product that may be evaluated in this article, or claim that may be made by its manufacturer, is not guaranteed or endorsed by the publisher.

## References

[1] World Health Organization. Building health systems resilience for universal health coverage and health security during the COVID-19 pandemic and beyond: WHO position paper. Geneva: WHO. (2021).

[2] Derek M Suan E. O. Introduction: COVID-19 and commonwealth countries. Introduction: COVID-19 and Commonwealth countries Common Wealth. J Int Aff. (2021) 110:10–5.

[3] WHO COVID-19 Dashboard. (2020). Available at: https://covid19.who.int/ (accessed 1 December 2022) Geneva: World health organization.

[4] Energising Commonwealth Trade in a Digital World. Commonwealth trade review 2021. Commonwealth Secretariat (2021).

[5] World Health Organization. Tracking Universal Health Coverage: 2021 global monitoring report. Geneva: World Health Organization and International Bank for Reconstruction and Development/The World Bank (2021).

[6] Communique from CHOGM. Commonwealth leaders travelled to Rwanda to reaffirm their common values and agree actions and policies to improve the lives of all their citizens. Rwanda: CHOGM (2022).

[7] Thomas S Sagan A Larkin J Cylus J Figueras J Karanikolos M. Strengthening health systems resilience: Key concepts and strategies. Copenhagen: European Observatory on Health Systems and Policies (2020). 32716618

[8] Department of Public Information. New COVID-19 response unit to be established – President Ali. Guyana: DPI (2020) dpi.gov.gy.

[9] Department of Public Information. Package of over 200 services drafted to improve primary healthcare. Government of Guyana. (2022). Available at: https://dpi.gov.gy/package-of-over-200-services-drafted-to-improve-primary-healthcare/ (Accessed January 15, 2023).

[10] World Health Organization. Joint external evaluation of IHR core capacities of the Republic of Malawi. Geneva: World health organization (2019) (WHO/WHE/CPI/2019.58). Licence: CC BY-NC-SA 3.0 IGO.

[11] Ministry of Health. Epidemiology and surveillance unit + MCH unit. COVID-19 vaccination summary. All data reported as of 30 November (2022).

[12] Gadabu A. Malawi’s response, risk factors, and preparedness for COVID-19. North Am Acad Res. (2020) 3:44–66.

[13] Village Reach. Impact evaluation of Chipatala cha PA Foni (CCPF). Lilongwe (2018). (accessed 24 Nov 2022).

[14] Republic of Rwanda. Data quality assessment procedures manual. Kigali: Ministry of Health (2016).

[15] World Health Organization (2018) Rwanda: Drones for community awareness and nation-wide measures in COVID-19 response (who.int) Coronavirus pandemic: applying a whole-of-society model for the whole-of-the world - Pub Med (nih.gov).

[16] Coronavirus Disease. Rwanda National Preparedness and Response Plan. Republic of Rwanda: Ministry of Public Health (2021).

[17] Liew MF Siow WT MacLaren G See KC. Preparing for COVID-19: early experience from an intensive care unit in Singapore. Crit Care. (2020) 24:83. doi: 10.1186/s13054-020-2814-x, PMID: 32151274 PMC7063757

[18] Wickramasinghe D Fernando VK. Sri Lanka’s fight against COVID-19: a brief overview. Pandemic Risk Resp Resil. (2022) 24:129–2. doi: 10.1016/B978-0-323-99277-0.00031-0

[19] Ministry of Health and Indigenous Medical Services (2020). Provisional clinical practice guidelines on COVID-19 suspected and confirmed patients Epidemiology unit, Ministry of Health and indigenous medical services Sri Lanka: Ministry of Health.

[20] Balabanova D McKee M Mills A. ‘Good health at low cost’ 25 years on. What makes a successful health system? London: London School of Hygiene and Tropical Medicine (2011).

[21] Ministry of Health. Performance and Progress Report. Sri Lanka: Ministry of Health (2021) 2022, October.

[22] Rajapaksa LC de Silva P Abeykoon P. COVID-19 health system response monitor: Sri Lanka. New Delhi: World Health Organization Regional Office for South-East Asia (2022).

[23] UNICEF. Maternal, child health, nutrition and immunization services in response to COVD 19 in Sri Lanka. New York: UNICEF (2020).

[24] Parodi SM Liu VX. From containment to mitigation of COVID-19 in the US. JAMA. (2020) 323:1441–2. doi: 10.1001/jama.2020.3882 32167525

[25] Grasselli G Pesenti A Cecconi M. Critical care utilization for the COVID-19 outbreak in Lombardy, Italy: early experience and forecast during an emergency response. JAMA. (2020) 323:1545–6. doi: 10.1001/jama.2020.4031 32167538

[26] World Health Organization. Progress report for the implementation of the COVID 19 response plan. Geneva: WHO Tanzania Country Office (2020).

[27] Kinkade C Russpatrick S Potter R Saebo J Sloan M Odongo G. Extending and strengthening routine DHIS2 surveillance systems for COVID-19 responses in Sierra Leone, Sri Lanka, and Uganda. Emerg Infect Dis. (2022) 28:42–8. doi: 10.3201/eid2813.220711 PMC9745217 36502427

[28] M&M. Digital Health Systems to Support Pandemic Response in Tanzania. Briefing. (2021).

[29] Mustafa U-k Kreppel KS Brinkel J Sauli E. Digital technologies to enhance infectious disease surveillance in Tanzania: a scoping review. Healthcare. (2023) 11:470. doi: 10.3390/healthcare11040470, PMID: 36833004 PMC9957254

[30] World Health Organization. Health systems resilience during COVID-19: lessons for building back better. World Health Organization Series (2021) 56 37023237

[31] Chen Z Cao C. Gonghuan Yang coordinated multi-sectoral efforts needed to address the COVID-19 pandemic: lessons from China and the United States. Glob Health Res Policy. (2020) 5:22 32391441 10.1186/s41256-020-00150-7 PMC7203075

[32] Haux R. Health information systems: past, present, future. Int J Med Inform. (2006) 75:268–1. doi: 10.1016/j.ijmedinf.2005.08.002 16169771

[33] WHO Regional Office for Europe. Strengthening and adjusting public health measures throughout the COVID-19 transition phases: Policy considerations for the WHO European region, 24 April 2020. Copenhagen: WHO Regional Office for Europe (2020).

[34] CDC, Using digital Technologies in Precision Public Health: COVID-19 and beyond. CDC (2020). Available at: https://blogs.cdc.gov/genomics/2020/04/06/using-digital/ (Accessed January 2023).

[35] World Health Organization (2020) Pulse survey on continuity of essential health services during the COVID-19 pandemic: interim report, 27 August 2020 (who.int).

[36] Webb E Quevedo CH Williams G Scarpetti G Reed S Panteli D. Providing health services effectively during the first wave of COVID-19: a cross-country comparison on planning services, managing cases, and maintaining essential services. Health Policy. (2022) 126:382–08. doi: 10.1016/j.healthpol.2021.04.016, PMID: 34246501 PMC8093167

[37] Saqif Mustafa Y Zhang ZZ Seifeldin R Ako-Egbe L McDarby G Saikat EKS. COVID-19 preparedness and response plans from 106 countries: a review from a health systems resilience perspective. Health Policy Plan. (2022) 37:255–8. doi: 10.1093/heapol/czab089, PMID: 34331439 PMC8385840

